# Impact of delivery time factor on treatment time and plan quality in tomotherapy

**DOI:** 10.1038/s41598-023-39047-z

**Published:** 2023-07-27

**Authors:** Takayuki Yagihashi, Tatsuya Inoue, Shintaro Shiba, Akihiro Yamano, Yumiko Minagawa, Motoko Omura, Kazumasa Inoue, Hironori Nagata

**Affiliations:** 1 Department of Medical Physics, Shonan Kamakura General Hospital, 1370-1 Okamoto, Kamakura, Kanagawa 247-8533 Japan; 2 Graduate School of Human Health Sciences, Tokyo Metropolitan University, 7-2-10 Higashiogu, Arakawa-ku, Tokyo 116-8551 Japan; 3 Department of Radiation Oncology, Graduate School of Medicine, Juntendo University, 2-1-1 Hongo, Bunkyo-ku, Tokyo 113-8421 Japan; 4 Department of Radiation Oncology, Shonan Kamakura General Hospital, 1370-1 Okamoto, Kamakura, Kanagawa 247-8533 Japan

**Keywords:** Radiotherapy, Lung cancer, Prostate cancer, Head and neck cancer

## Abstract

Delivery time factor (DTF) is a new parameter introduced by the RayStation treatment planning system for tomotherapy treatment planning. This study investigated the effects of this factor on various tomotherapy plans. Twenty-five patients with cancer (head and neck, 6; lung, 9; prostate, 10) were enrolled in this study. Helical tomotherapy plans with a field width of 2.5 cm, pitch of 0.287, and DTF of 2.0 were created. All the initial plans were recalculated by changing the DTF parameter from 1.0 to 3.0 in increments of 0.1. Then, DTF’s impact on delivery efficiency and plan quality was evaluated. Treatment time and modulation factor increased monotonically with increasing DTF. Increasing the DTF by 0.1 increased the treatment time and modulation factor by almost 10%. This relationship was similar for all treatment sites. Conformity index (CI), homogeneity index, and organ at risk doses were improved compared to plans with a DTF of 1.0, except for the CI in the lung cancer case. However, the improvement in most indices ceased at a certain DTF; nevertheless, treatment time continued to increase following an increase in DTF. DTF is a critical parameter for improving the quality of tomotherapy plans.

## Introduction

Radixact (Accuray Inc., Sunnyvale, CA, USA) is the latest tomotherapy radiation therapy delivery unit capable of delivering intensity-modulated radiation therapy (IMRT)^1,2^. The unit uses a flattening filter-free 6 MV linear accelerator mounted on a slip-ring-gantry that moves in synchronization with a treatment couch and has improved the treatment time efficiency with respect to imaging and delivery compared to previous versions. On the Radixact, an x-ray tube and flat-panel kV imager are equipped in addition to a conventional megavolt computed tomography (MVCT) system, and a helical kVCT system is available for clinical use as an optional add-on. These systems can be used for pretreatment patient setup verification, dose recalculation, and patient and machine quality assurance^3–5^. The machine enables the delivery of a highly conformal dose to complex target volumes while minimizing the dose to critical normal tissues by slice-by-slice delivery with 64 binary multileaf collimators. Many studies have demonstrated that tomotherapy is suitable for the treatment of a wide spectrum of anatomical sites, including the brain^6,7^, head and neck^8–10^, lung^11–13^, breast^14,15^, abdomen and pelvis^16–18^, and prostate^19–21^. Moreover, due to the unique feature capable of irradiating long targets up to 130 cm, it is effective for treatments such as total body irradiation, total marrow irradiation, and craniospinal irradiation^22–25^.

Treatment plan quality depends on the performance of the optimizer and the selection of appropriate planning parameters in the treatment planning system (TPS). Tomotherapy has specific planning parameters such as field width (FW), pitch, and modulation factor (MF), which can affect the quality of plans and treatment times. The FW is defined as the longitudinal thickness of the fan beam. Pitch is defined as the couch travel distance for a complete gantry rotation relative to the axial beam width at the axis of rotation. Generally, a pitch of 0.86/n (n is an integer) is used to mitigate the thread effect^26,27^. The MF is defined as the maximum leaf opening time divided by the average (non-zero) leaf opening time and is an estimate of plan complexity^28,29^. Increasing the MF enables larger beam modulation resulting in high-quality plans; however, this leads to increased treatment time^2,30^. In clinical practice, the selection of these parameters is unique to the treatment site and represents a compromise between longer treatment times with superior dose distribution and shorter treatment times with poorer dose distribution. Consequently, planning is subject to the planner’s experience, and the created plans can be biased^31^.

RayStation TPS (RaySearch Lab; Stockholm, Sweden) has recently implemented a dose calculation module for tomotherapy. In the optimization process, three different types of delivery time constraints are supported: (1) delivery time factor (DTF), (2) maximum gantry period, and (3) maximum delivery time, while the MF is unavailable^32^. A DTF of *x* is a factor to extend the delivery time *t*, where *t* means the time required to irradiate the prescription dose to the target uniformly at the DTF of 1.0. For instance, the value of 2.0 means two times of delivery time is used for the treatment compared to 1.0. In other words, increasing the DTF leads to the extension of the opening time of multileaf collimators throughout the treatment, resulting in higher beam modulation. As this factor is associated with multileaf collimators, it is expected to be related to delivery efficiency and plan quality as is the case with MF. Thus, understanding the DTF’s impact on treatment planning could influence the decision to adjust it to reduce the number of trial-and-error attempts during planning. However, to the best of our knowledge, no study has investigated the impacts of the use of DTF in treatment planning.

This study aimed to evaluate the impact of changes in DTF in helical tomotherapy plans for head and neck, lung, and prostate cancers on target dose conformity and homogeneity, organs at risk (OARs) doses, and treatment time.

## Methods

Twenty-five patients previously treated with helical tomotherapy, which was planned using tomotherapy-dedicated TPS Precision (Accuray, Inc., Madison, Wisconsin, USA) between January 2021 and July 2022 at Shonan Kamakura General Hospital, were enrolled. The population consisted of (1) six patients with head and neck cancer treated with simultaneous integrated boost for primary tumor and macroscopic suspicious cervical lymph nodes, (2) nine patients with stage III non-small cell lung cancer, and (3) ten patients with prostate cancer with intermediate-risk. The patient characteristics are summarized in Table 1.

**Table 1:** Patient characteristics.

Patient	Treatment site	Age	cTNM stage	PTV (cc)	Primary tumor location
H01	Head & neck	62	T2N1M0	248.0	
H02		77	T2N0M0	46.6	
H03		78	T3N2bM0	225.7	
H04		80	T4aN3M0	421.3	
H05		78	T4aN0M0	165.4	
H06		60	T3N2cM0	599.5	
L01	Lung	78	T4N0M0	721.1	Left upper lobe
L02		73	T1N3M0	385.5	Right upper lobe
L03		82	T3N2M0	311.6	Mediastinum
L04		81	T3N2M0	222.7	Left lower lobe
L05		69	TxN3M0	129.0	Mediastinum
L06		74	T2aN3M0	347.3	Right lower lobe
L07		66	T2bN2M0	175.5	Left upper lobe
L08		76	T1bN2M0	98.6	Left upper lobe
L09		68	T2N2M0	107.3	Right lower lobe
P01	Prostate	87	T2cN0M0	77.4	
P02		85	T1cN0M0	74.7	
P03		73	T2cN0M0	71.1	
P04		87	T2bN0M0	142.3	
P05		76	T2aN0M0	75.4	
P06		76	T1cN0M0	77.3	
P07		79	T1cN0M0	135.8	
P08		71	T3aN0M0	80.6	
P09		57	T3aN0M0	70.6	
P10		75	T1cN0M0	78.2	

### Structure contouring

All CT images were obtained using a Siemens 20-slice CT scanner (Somatom Confidence, Siemens, Germany) with a reconstruction resolution of 0.967 × 0.967 × 2 mm^3^. Radiation oncologists with more than 10 years of experience delineated the target volume and representative OARs using RayStation version 10A.

- Head and neck cancer

The gross tumor volume (GTV), the high-risk clinical target volume (CTV) involved 5 mm expansion from the GTV; intermediate-risk CTV involved high-risk CTV with at-risk lymphatic drainage in the node-positive region; and low-risk CTV involved low-risk lymphatic regions were delineated. The corresponding planning target volumes (PTV_low, PTV_intermediate, and PTV_high) were created by adding an isotropic 5 mm margin to the CTVs. The PTV volumes in Table 1 are the PTV_high volumes. Both parotid glands, the brainstem, and the spinal cord were defined as the OARs.

(b) Lung cancer

The internal target volume (ITV) was defined as the union of GTVs in 10 phases of four-dimensional CTs. The CTV was created by the expansion of the ITV by a 5 mm margin in all directions, and non-invasive and bone regions were excluded from the CTV. The PTV was created by the expansion of the CTV by a 5 mm margin. Both lungs, esophagus, heart, and spinal cord were defined as OARs. These delineations were consequently performed on maximum inspiration CT.

(c) Prostate cancer

The CTV was defined as the prostate and the proximal seminal vesicles. The PTV was created by adding 8 mm margins in each direction to the CTV, except for the posterior direction of a 5 mm margin. The bladder and rectum were defined as OARs. The contouring details for prostate cancer have been previously described^33^.

### Treatment planning

Treatment planning was performed by a medical physicist using the RayStation commissioned for Radixact. First, helical tomotherapy plans were optimized using a FW of 2.5 cm, pitch of 0.287, and a DTF of 2.0. For head and neck plans, the prescription doses were 77, 70, and 56 Gy in 35 fractions for PTV_high, PTV_intermediate, and PTV_low, respectively^34^. The dose was normalized using 95% of the PTV_high with 77 Gy. For lung plans, the prescription dose was 66 Gy in 33 fractions. The dose was normalized using 99% of the PTV with 95% of the prescription dose^35^. For prostate plans, the prescription dose was 60 Gy in 30 fractions. The dose was normalized using 95% of the PTV with the prescription dose^33,36^. The dose objectives needed to be achieved for the target volumes and OARs in each treatment planning are shown in Table 2. In addition, the dose fall-off option was used for the volume up to 0.3 cm and from 0.3 to 0.8 cm outside the PTV to control the dose fall-off for the volume outside the PTV. The objective weights were varied to spare the OAR doses as much as possible without compromising the target criteria. All plans were optimized using a dose grid size of 2 mm and 60 iterations, with the final calculation every 20 iterations. After creating the initial plans, all plans were recalculated by changing the DTF parameter from 1.0 to 3.0 in increments of 0.1. The planning and optimization parameters, except for DTF, were kept identical to the initial plans for fair comparison purposes. Totally, 525 treatment plans (21 per patient) were included in this study.

**Table 2:** Dose objectives for treatment planning.

Head and neck		Lung		Prostate	
Target volume	Dose objective	Target volume	Dose objective	Target volume	Dose objective
PTV_high	D_95%_ = 77 Gy	PTV	V_95%_ > 99%	PTV	D_95%_ = 60 Gy
	D_50%_ < 79.3 Gy		D_max_ < 64.2 Gy		D_max_ < 64.2 Gy
	D_max_ < 82.4 Gy				
PTV_intermediate	D_95%_ = 70 Gy				
	D_50%_ < 72.1 Gy				
	D_max_ < 74.9 Gy				
PTV_low	D_95%_ = 56 Gy				
	D_50%_ < 57.7 Gy				
	D_max_ < 59.9Gy				

Organ at risk	Dose objective	Organ at risk	Dose objective	Organ at risk	Dose objective
Spinal cord	D_max_ < 50 Gy	Lungs	D_mean_ < 20 Gy	Rectum	V_30 Gy_ < 50%
	1 cc < 45 Gy		V_20 Gy_ < 30%		V_57 Gy_ < 15%
Brainstem	D_max_ < 54 Gy	Esopagus	V_65 Gy_ < 35%	Bladder	V_40 Gy_ < 50%
	1 cc < 54 Gy		D_max_ < 66 Gy		V_50 Gy_ < 30%
Mandible	D_max_ < 70 Gy	Spinal cord	D_max_ < 50 Gy		V_60 Gy_ < 5%
	1 cc < 70 Gy	Heart	V_50 Gy_ < 40%		
Parotid glands	D_mean_ < 26 Gy				
	D_50%_ < 30 Gy				
	20 cc of both < 20 Gy				

### Data reporting and analysis

The Paddick conformity index (CI)^37^ and homogeneity index (HI)^38^ were calculated for all plans to evaluate the change in the quality of the plan with changes in the DTF parameter. The CI was calculated using Eq. (1):

1 $$\mathrm{CI}= \frac{{\mathrm{TV}}_{\mathrm{PIV}}^{2}}{\left(\mathrm{TV}\times {\mathrm{V}}_{\mathrm{RI}}\right)},$$

where TV is the target volume, TV_PIV_ is the target volume covered by the prescribed dose, and V_RI_ is the total volume covered by the prescribed dose. The HI was calculated using Eq. (2):

2 $$\mathrm{HI}= \frac{{\mathrm{D}}_{2\mathrm{\%}}-{\mathrm{D}}_{98\mathrm{\%}}}{{\mathrm{D}}_{50\mathrm{\%}}},$$

where D_2%_, D_98%_, and D_50%_ are the doses covering 2, 98, and 50% of the target volume, respectively. Values close to 1 and 0 are the ideal values for the CI and HI, respectively. For the head and neck plans, these indices were calculated for PTV_high. For OARs, the maximum dose, defined as D_2%_ or the mean dose, was calculated. Notably, dose evaluation for the mandible was not performed in this study. The treatment time, MF, and summed residual objective value for each plan were also gathered to evaluate the delivery efficiency.

Then, to investigate the effect of the DTF parameter, changing rates of evaluation indices normalized by the corresponding indices at the DTF of 1.0 between DTF *x*-0.1 and DTF *x* (*x* ranges from 1.1 to 3.0) were calculated as “Δ”. Furthermore, the accumulation values of the changing rates from a DTF of 1.1 to each DTF (up to 3.0) were calculated as “Accum”.

### Ethics approval and consent to participate

This study was approved by the institutional review board of Shonan Kamakura General Hospital (The Tokushukai Group Ethics Committee, No. 2035). All methods were performed in accordance with the relevant guidelines and regulations. Given its retrospective nature, the review board waived the need for informed consent by offering an opt-out option on the institution’s homepage (The Tokushukai Group Ethics Committee, https://www.mirai-iryo.com/service/index.php#s03).

## Results

Table 3 lists the mean values and standard deviations of the treatment time, MF, CI, HI, and OAR indices for each DTF plan.

**Table 3:** Evaluation indices (means and standard deviations) for treatment plans with a DTF from 1.0 to 3.0.

Head & neck			DTF																				
			1.0	1.1	1.2	1.3	1.4	1.5	1.6	1.7	1.8	1.9	2.0	2.1	2.2	2.3	2.4	2.5	2.6	2.7	2.8	2.9	3.0
Time	[sec]	Mean	351.5	384.2	419.8	455.7	490.3	526.0	558.7	595.2	630.2	666.0	702.7	735.0	770.5	806.2	841.3	877.2	910.2	945.7	979.7	1014.5	1048.7
		SD	48.8	52.9	57.5	63.2	68.0	71.9	76.0	82.3	86.8	91.0	96.5	100.4	105.5	110.9	115.8	120.1	125.2	130.1	133.4	135.4	138.8
MF		Mean	2.44	2.67	2.93	3.17	3.43	3.69	3.92	4.18	4.43	4.68	4.94	5.17	5.42	5.67	5.92	6.17	6.39	6.65	6.89	7.13	7.38
		SD	0.11	0.12	0.13	0.14	0.16	0.17	0.17	0.18	0.19	0.20	0.21	0.21	0.22	0.23	0.24	0.25	0.25	0.27	0.29	0.30	0.32
CI		Mean	0.84	0.84	0.85	0.85	0.85	0.85	0.85	0.85	0.85	0.85	0.85	0.85	0.85	0.85	0.85	0.85	0.85	0.85	0.85	0.85	0.85
		SD	0.03	0.03	0.03	0.03	0.03	0.03	0.03	0.03	0.03	0.03	0.03	0.03	0.03	0.03	0.03	0.03	0.03	0.03	0.03	0.03	0.03
HI		Mean	0.080	0.078	0.077	0.076	0.076	0.075	0.075	0.075	0.075	0.075	0.075	0.076	0.076	0.076	0.076	0.076	0.076	0.076	0.075	0.075	0.075
		SD	0.017	0.015	0.014	0.014	0.014	0.014	0.014	0.014	0.014	0.014	0.014	0.014	0.014	0.014	0.014	0.015	0.015	0.014	0.015	0.014	0.014
Parotid glands D_mean_	[Gy]	Mean	14.7	14.5	14.4	14.2	14.1	14.1	14.0	13.9	13.9	13.9	13.8	13.8	13.8	13.7	13.7	13.7	13.7	13.7	13.6	13.6	13.6
		SD	4.7	4.7	4.7	4.6	4.5	4.5	4.5	4.5	4.5	4.4	4.5	4.5	4.4	4.4	4.4	4.4	4.4	4.4	4.4	4.4	4.4
Brainstem D_max_	[Gy]	Mean	21.2	21.0	21.0	21.1	21.3	21.2	21.2	21.2	21.3	21.4	21.2	21.2	21.0	20.9	20.9	21.0	21.0	21.0	20.9	20.9	20.9
		SD	16.5	16.4	16.3	16.5	16.5	16.6	16.6	16.5	16.6	16.8	16.8	16.8	16.6	16.7	16.6	16.6	16.7	16.7	16.7	16.7	16.7
Cord D_max_	[Gy]	Mean	41.9	41.6	41.7	41.8	41.7	41.7	41.8	42.0	42.0	42.0	42.2	42.1	42.2	42.3	42.2	42.3	42.3	42.2	42.2	42.3	42.3
		SD	4.0	4.0	4.2	3.9	4.1	4.1	4.1	4.1	4.0	3.9	3.8	3.9	3.9	4.0	4.0	3.9	3.9	3.9	3.9	3.8	3.9

Lung			DTF																				
			1.0	1.1	1.2	1.3	1.4	1.5	1.6	1.7	1.8	1.9	2.0	2.1	2.2	2.3	2.4	2.5	2.6	2.7	2.8	2.9	3.0
Time	[sec]	Mean	266.2	290.1	316.1	342.2	368.9	395.8	420.2	446.9	473.6	499.4	526.2	551.0	577.1	602.6	629.2	655.3	679.9	704.8	729.6	754.8	781.1
		SD	85.3	93.5	101.6	109.9	118.6	127.1	134.8	143.4	151.9	159.5	169.1	177.5	184.9	191.0	198.5	206.0	214.0	220.2	226.9	235.8	241.0
MF		Mean	1.88	2.05	2.26	2.46	2.66	2.87	3.06	3.25	3.46	3.65	3.85	4.03	4.22	4.41	4.60	4.80	4.98	5.16	5.35	5.54	5.72
		SD	0.26	0.27	0.29	0.31	0.34	0.36	0.38	0.40	0.43	0.45	0.47	0.49	0.50	0.52	0.54	0.57	0.59	0.61	0.63	0.65	0.67
CI		Mean	0.67	0.67	0.67	0.63	0.60	0.64	0.62	0.62	0.59	0.61	0.58	0.58	0.57	0.59	0.60	0.59	0.60	0.60	0.59	0.60	0.58
		SD	0.13	0.14	0.14	0.16	0.17	0.14	0.16	0.16	0.17	0.16	0.16	0.17	0.17	0.15	0.14	0.15	0.15	0.15	0.15	0.14	0.15
HI		Mean	0.075	0.071	0.069	0.068	0.067	0.068	0.067	0.067	0.067	0.067	0.067	0.067	0.067	0.067	0.067	0.067	0.067	0.067	0.067	0.068	0.067
		SD	0.012	0.010	0.007	0.006	0.005	0.005	0.005	0.005	0.005	0.005	0.005	0.005	0.005	0.005	0.005	0.005	0.005	0.005	0.005	0.005	0.005
Lung D_mean_	[Gy]	Mean	12.9	12.8	12.7	12.6	12.6	12.6	12.6	12.6	12.6	12.6	12.6	12.6	12.6	12.6	12.6	12.6	12.6	12.6	12.5	12.5	12.5
		SD	4.0	3.8	3.8	3.7	3.7	3.7	3.7	3.7	3.7	3.7	3.7	3.7	3.7	3.7	3.7	3.7	3.7	3.7	3.7	3.7	3.7
Esophagus D_mean_	[Gy]	Mean	21.0	20.9	20.9	20.8	20.7	20.7	20.7	20.7	20.7	20.7	20.7	20.7	20.6	20.6	20.6	20.7	20.7	20.7	20.7	20.7	20.6
		SD	11.7	11.7	11.7	11.7	11.7	11.7	11.6	11.6	11.6	11.6	11.6	11.6	11.6	11.6	11.6	11.6	11.6	11.6	11.6	11.6	11.6
Cord D_max_	[Gy]	Mean	40.6	40.4	40.3	40.2	40.2	40.1	40.0	40.0	40.0	39.9	39.9	39.8	39.8	39.8	39.8	39.8	39.8	39.8	39.8	39.8	39.8
		SD	11.2	11.3	11.3	11.4	11.5	11.6	11.6	11.7	11.7	11.7	11.7	11.7	11.7	11.7	11.7	11.7	11.8	11.8	11.7	11.7	11.7
Heart D_mean_	[Gy]	Mean	15.1	15.1	15.0	15.0	15.0	15.0	15.0	14.9	14.9	14.9	14.9	14.9	14.9	14.9	14.9	14.9	14.9	14.9	14.9	14.9	14.9
		SD	8.5	8.4	8.4	8.4	8.4	8.3	8.3	8.3	8.3	8.3	8.3	8.3	8.3	8.3	8.3	8.3	8.3	8.3	8.3	8.3	8.3

Prostate			DTF																				
			1.0	1.1	1.2	1.3	1.4	1.5	1.6	1.7	1.8	1.9	2.0	2.1	2.2	2.3	2.4	2.5	2.6	2.7	2.8	2.9	3.0
Time	[sec]	Mean	149.6	163.0	177.6	192.5	207.4	222.2	234.6	249.2	265.9	281.8	296.8	310.3	324.2	339.0	354.9	369.3	382.2	397.9	413.2	427.5	442.1
		SD	13.7	14.6	15.9	17.4	19.1	20.2	21.6	22.8	24.2	26.8	28.5	29.3	29.8	30.9	33.2	33.5	35.5	38.8	40.0	42.2	42.5
MF		Mean	1.17	1.28	1.39	1.51	1.64	1.77	1.89	2.02	2.15	2.29	2.42	2.54	2.67	2.80	2.94	3.07	3.19	3.33	3.46	3.59	3.72
		SD	0.05	0.05	0.07	0.08	0.09	0.10	0.10	0.12	0.12	0.14	0.15	0.15	0.16	0.16	0.16	0.17	0.17	0.17	0.18	0.18	0.19
CI		Mean	0.82	0.82	0.82	0.82	0.83	0.83	0.84	0.84	0.84	0.84	0.84	0.83	0.83	0.83	0.83	0.83	0.83	0.83	0.83	0.82	0.82
		SD	0.03	0.02	0.02	0.02	0.02	0.03	0.02	0.02	0.02	0.02	0.02	0.02	0.02	0.02	0.02	0.02	0.02	0.02	0.02	0.02	0.02
HI		Mean	0.084	0.075	0.070	0.068	0.066	0.064	0.063	0.062	0.062	0.062	0.063	0.063	0.064	0.064	0.064	0.065	0.065	0.066	0.066	0.067	0.067
		SD	0.012	0.008	0.005	0.004	0.004	0.004	0.006	0.005	0.005	0.005	0.005	0.005	0.004	0.005	0.005	0.005	0.005	0.005	0.005	0.005	0.005
Bladdr D_mean_	[Gy]	Mean	19.2	18.8	18.5	18.4	18.3	18.2	18.1	18.0	18.0	17.9	17.9	17.9	17.9	17.9	17.8	17.9	17.8	17.8	17.9	17.9	17.8
		SD	5.9	5.7	5.5	5.5	5.5	5.4	5.4	5.3	5.3	5.3	5.3	5.3	5.3	5.2	5.2	5.2	5.2	5.2	5.2	5.2	5.2
Rectum D_mean_	[Gy]	Mean	22.6	21.5	20.9	20.5	20.3	20.1	20.0	20.0	19.9	19.8	19.8	19.8	19.8	19.7	19.7	19.8	19.7	19.7	19.8	19.8	19.8
		SD	5.8	5.6	5.5	5.5	5.5	5.5	5.5	5.5	5.4	5.4	5.4	5.4	5.4	5.4	5.4	5.4	5.4	5.4	5.4	5.4	5.4

**Figure 1:**
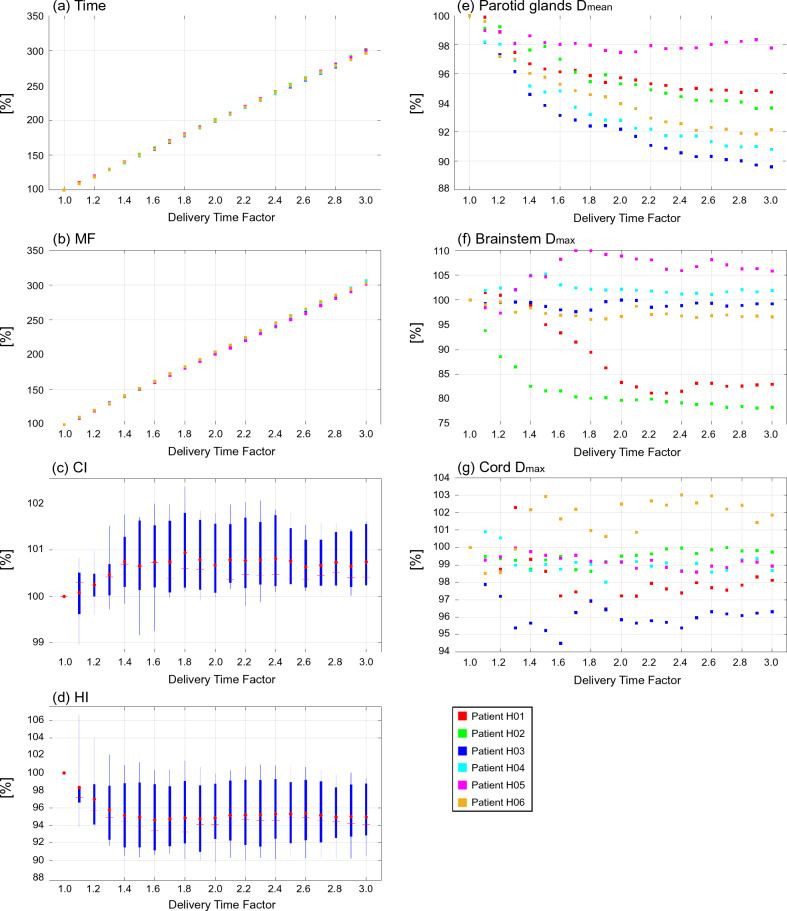
Plot diagrams and boxplots of evaluation indices normalized by the indices at the DTF value of 1.0 for plans with a DTF ranging from 1.0 to 3.0 in six patients with head and neck cancer. (**a**) treatment time [Time], (**b**) modulation factor [MF], (**c**) conformity index [CI], (**d**) homogeneity index [HI], (**e**) mean doses to both parotid glands [Parotid glands D_mean_], (**f**) maximum doses to brainstem [Brainstem D_max_], and (**g**) maximum doses to cord [Cord D_max_]. Each box in the boxplots comprises the minimum and maximum range values, upper and lower quartiles, and average (red circle) and median (red line) values.

**Figure 2:**
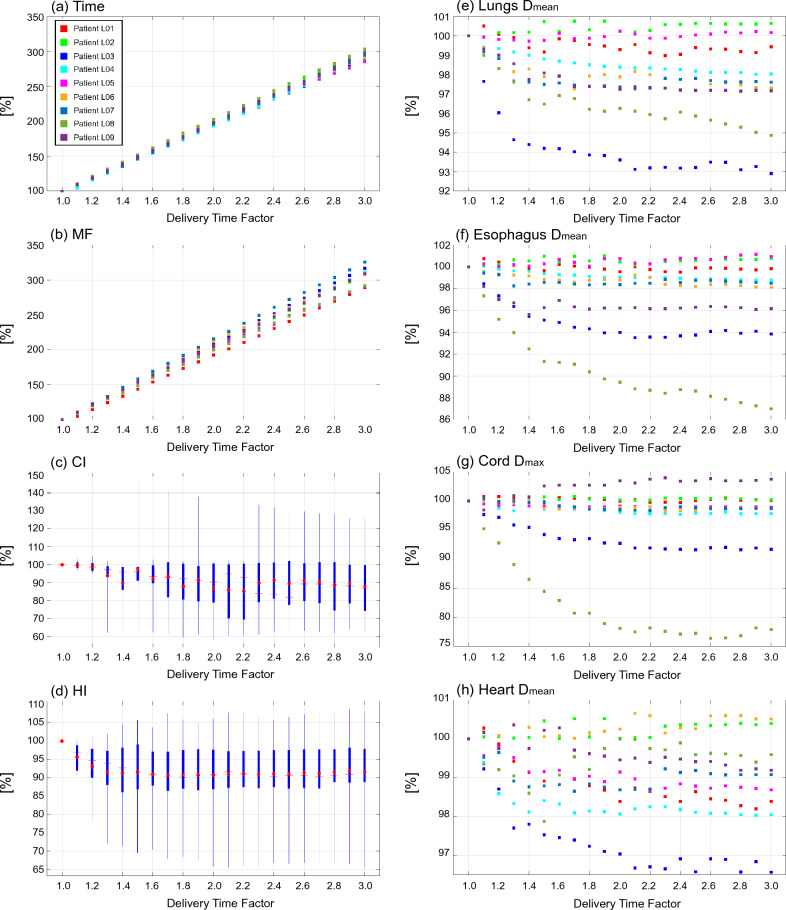
Plot diagrams and boxplots of evaluation indices normalized by the indices at the DTF value of 1.0 for plans with a DTF ranging from 1.0 to 3.0 in nine patients with lung cancer. (**a**) treatment time [Time], (**b**) modulation factor [MF], (**c**) conformity index [CI], (**d**) homogeneity index [HI], (**e**) mean doses to lungs [Lungs D_mean_], (**f**) mean doses to esophagus [Esophagus D_mean_], (**g**) maximum doses to cord [Cord D_max_], and (**h**) mean doses to heart [Heart D_mean_]. Each box in the boxplots comprises the minimum and maximum range values, upper and lower quartiles, and average (red circle) and median (red line) values.

**Figure 3:**
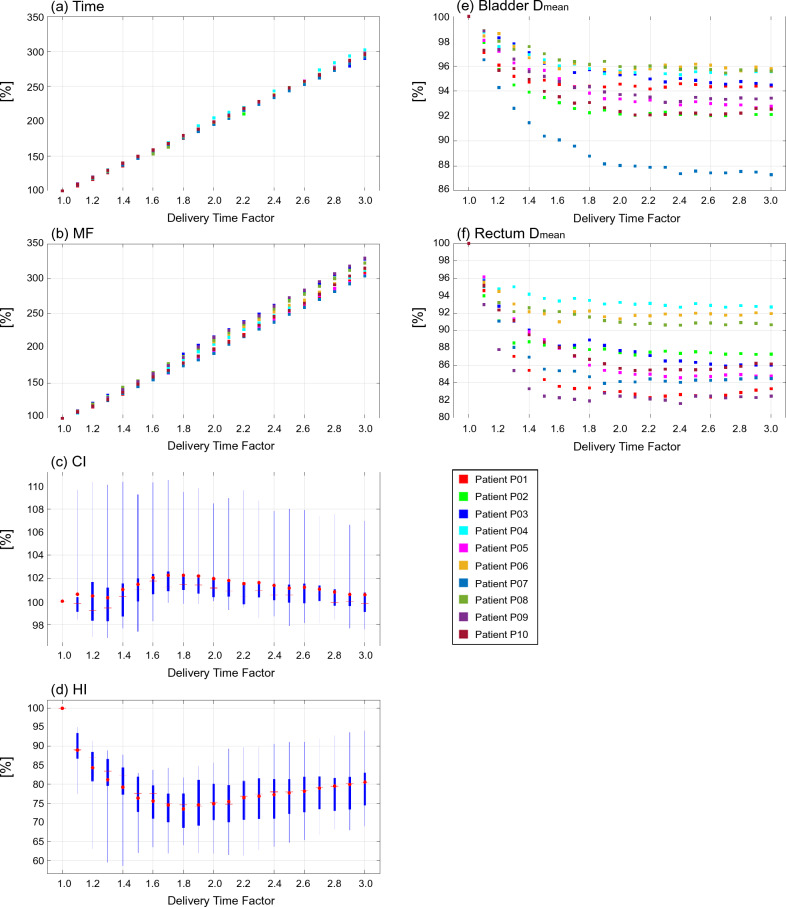
Plot diagrams and boxplots of evaluation indices normalized by the indices at the DTF value of 1.0 for plans with a DTF ranging from 1.0 to 3.0 in 10 patients with prostate cancer. (**a**) treatment time [Time], (**b**) modulation factor [MF], (**c**) conformity index [CI], (**d**) homogeneity index [HI], (**e**) mean doses to bladder [Bladder D_mean_], (**f**) mean doses to rectum [Rectum D_mean_]. Each box in the boxplots comprises the minimum and maximum range values, upper and lower quartiles, and average (red circle) and median (red line) values.

Table S1 summarizes the summed residual objective values for all DTF plans. The results demonstrated that the values did not completely achieve 0 in all DTF plans. Figures 1, 2 and 3 show diagrams that plot every evaluation index normalized by the corresponding indices of the plans with a DTF of 1.0.

**Figure 4:**
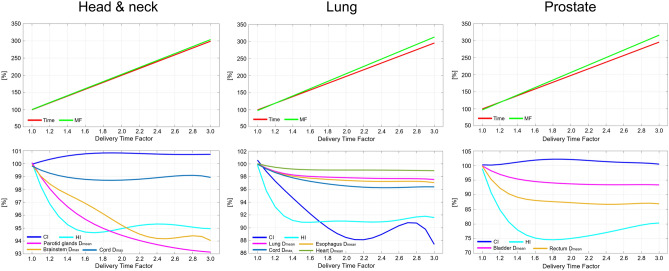
Fitted curves of evaluation indices as a function of delivery time factor in head & neck, lung, and prostate cancer sites. Top figures show the curves for treatment time [Time] and modulation factor [MF], bottom figures show the curves for conformity index [CI] and homogeneity index [HI] for the targets, and organ at risk indices.

Figure 4 shows fitted curves as a function of the DTF for each index. The curves that fit the plot data in Figs. 1, 2 and 3 were created using a first-order polynomial for the treatment time and MF and a fifth-order polynomial for the CI, HI, and OAR indices.

Table 4 summarizes the rate of change for each index on the fitted curves. The change of each normalized index between DTF *x*-0.1 and DTF *x* (*x* ranges from 1.1 to 3.0) is denoted “Δ”. Accum is the accumulation of the changing rates from a DTF of 1.1 to the corresponding DTF. The treatment time demonstrated an approximately linear correlation with DTF. The treatment time increased by almost 200% when using a DTF of 3.0 than when using a DTF of 1.0. The MF also exhibited the same trend as treatment time. In head and neck and prostate cancers, the CI improved by 0.9% and 1.9% when the DTF was 1.9 and 1.8, respectively, and did not improve thereafter. In contrast, the CI exhibited the best value when the DTF was 1.0 in lung cancer. In terms of HI, an increase in the DTF initially improved the index by 5.4% (head and neck cancer case; DTF 1.7), 9.0% (lung cancer case; DTF 1.6), and 1.9% (prostate cancer case; DTF 1.8), after which the improvement plateaued. The doses for both parotid glands in cases with head and neck cancer continued to decrease until a DTF of 3.0 was applied, and the rates of decrease were 6.9%. The maximum dose to the brainstem and cord decreased with an increase in DTF up to 2.4 and 1.7, and the decrease plateaued at higher DTF values. The decrease in the OAR doses for lung cases plateaued when the DTF was in the range of 1.5-2.3. The bladder and rectum doses were the lowest when the plan was created using a DTF of 2.4, and their doses decreased by 6.5% and 13.1%, respectively.

**Table 4:** Rate of change (Δ) and the accumulation (Accum) of evaluation indices on the fitted curves with changes in DTF.

Head & neck		DTF																			
		1.1	1.2	1.3	1.4	1.5	1.6	1.7	1.8	1.9	2.0	2.1	2.2	2.3	2.4	2.5	2.6	2.7	2.8	2.9	3.0
Time	Δ [%]	10.0	10.0	10.0	10.0	10.0	10.0	10.0	10.0	10.0	10.0	10.0	10.0	10.0	10.0	10.0	10.0	10.0	10.0	10.0	10.0
	Accum [%]	10.0	19.9	29.9	39.8	49.8	59.8	69.7	79.7	89.6	99.6	109.6	119.5	129.5	139.5	149.4	159.4	169.3	179.3	189.3	199.2
MF	Δ [%]	10.2	10.2	10.2	10.2	10.2	10.2	10.2	10.2	10.2	10.2	10.2	10.2	10.2	10.2	10.2	10.2	10.2	10.2	10.2	10.2
	Accum [%]	10.2	20.3	30.5	40.7	50.8	61.0	71.2	81.3	91.5	101.7	111.8	122.0	132.2	142.3	152.5	162.7	172.9	183.0	193.2	203.4
CI	Δ [%]	0.2	0.2	0.1	0.1	0.1	0.1	0.0	0.0	0.0	0.0	0.0	0.0	0.0	0.0	0.0	0.0	0.0	0.0	0.0	0.0
	Accum [%]	0.2	0.4	0.5	0.7	0.7	0.8	0.8	0.9	0.9	0.9	0.9	0.8	0.8	0.8	0.8	0.8	0.8	0.8	0.8	0.8
HI	Δ [%]	− 1.8	− 1.4	− 1.0	− 0.6	− 0.4	− 0.2	0.0	0.1	0.1	0.1	0.1	0.1	0.1	0.0	0.0	− 0.1	− 0.1	− 0.1	− 0.1	0.0
	Accum [%]	− 1.8	− 3.2	− 4.2	− 4.8	− 5.2	− 5.4	− 5.4	− 5.4	− 5.3	− 5.1	− 5.0	− 4.9	− 4.8	− 4.8	− 4.8	− 4.8	− 4.9	− 5.0	− 5.1	− 5.1
Parotid glands D_mean_	Δ [%]	− 1.1	− 0.9	− 0.8	− 0.6	− 0.5	− 0.4	− 0.4	− 0.3	− 0.3	− 0.2	− 0.2	− 0.2	− 0.2	− 0.2	− 0.1	− 0.1	− 0.1	− 0.1	− 0.1	− 0.1
	Accum [%]	− 1.1	− 2.0	− 2.8	− 3.4	− 3.9	− 4.4	− 4.8	− 5.1	− 5.4	− 5.6	− 5.8	− 6.0	− 6.2	− 6.3	− 6.5	− 6.6	− 6.7	− 6.8	− 6.9	− 6.9
Brainstem D_max_	Δ [%]	− 0.9	− 0.6	− 0.4	− 0.4	− 0.3	− 0.4	− 0.4	− 0.4	− 0.4	− 0.4	− 0.4	− 0.3	− 0.2	− 0.1	0.0	0.1	0.1	0.1	− 0.1	− 0.3
	Accum [%]	− 0.9	− 1.5	− 1.9	− 2.3	− 2.6	− 3.0	− 3.4	− 3.8	− 4.2	− 4.7	− 5.0	− 5.4	− 5.6	− 5.7	− 5.7	− 5.7	− 5.6	− 5.5	− 5.6	− 5.9
Cord D_max_	Δ [%]	− 0.3	− 0.2	− 0.2	− 0.1	− 0.1	− 0.1	0.0	0.0	0.0	0.0	0.0	0.0	0.1	0.1	0.1	0.1	0.0	0.0	0.0	− 0.1
	Accum [%]	− 0.3	− 0.6	− 0.7	− 0.9	− 1.0	− 1.0	− 1.1	− 1.1	− 1.1	− 1.1	− 1.1	− 1.0	− 1.0	− 0.9	− 0.8	− 0.8	− 0.7	− 0.7	− 0.8	− 0.9

Lung		DTF																			
		1.1	1.2	1.3	1.4	1.5	1.6	1.7	1.8	1.9	2.0	2.1	2.2	2.3	2.4	2.5	2.6	2.7	2.8	2.9	3.0
Time	Δ [%]	9.7	9.7	9.7	9.7	9.7	9.7	9.7	9.7	9.7	9.7	9.7	9.7	9.7	9.7	9.7	9.7	9.7	9.7	9.7	9.7
	Accum [%]	9.7	19.5	29.2	39.0	48.7	58.5	68.2	78.0	87.7	97.5	107.2	117.0	126.7	136.5	146.2	156.0	165.7	175.5	185.2	195.0
MF	Δ [%]	10.3	10.3	10.3	10.3	10.3	10.3	10.3	10.3	10.3	10.3	10.3	10.3	10.3	10.3	10.3	10.3	10.3	10.3	10.3	10.3
	Accum [%]	10.3	20.6	30.9	41.2	51.6	61.9	72.2	82.5	92.8	103.1	113.4	123.7	134.0	144.4	154.7	165.0	175.3	185.6	195.9	206.2
CI	Δ [%]	− 1.8	− 1.5	− 1.4	− 1.3	− 1.3	− 1.2	− 1.2	− 1.0	− 0.9	− 0.6	− 0.3	0.0	0.3	0.5	0.7	0.7	0.5	− 0.1	− 1.0	− 2.4
	Accum [%]	− 1.8	− 3.3	− 4.6	− 5.9	− 7.2	− 8.4	− 9.6	− 10.6	− 11.5	− 12.1	− 12.4	− 12.5	− 12.2	− 11.7	− 11.0	− 10.3	− 9.8	− 9.9	− 10.8	− 13.2
HI	Δ [%]	− 4.0	− 2.5	− 1.4	− 0.7	− 0.3	0.0	0.1	0.1	0.0	0.0	− 0.1	− 0.1	0.0	0.0	0.1	0.2	0.2	0.2	0.1	− 0.2
	Accum [%]	− 4.0	− 6.6	− 8.0	− 8.7	− 9.0	− 9.0	− 8.9	− 8.8	− 8.8	− 8.8	− 8.9	− 8.9	− 8.9	− 8.9	− 8.8	− 8.6	− 8.4	− 8.1	− 8.0	− 8.2
Lung D_mean_	Δ [%]	− 0.7	− 0.5	− 0.3	− 0.2	− 0.1	− 0.1	− 0.1	0.0	0.0	0.0	0.0	0.0	0.0	0.0	0.0	0.0	0.0	0.0	0.0	− 0.1
	Accum [%]	− 0.7	− 1.2	− 1.6	− 1.8	− 1.9	− 2.0	− 2.1	− 2.1	− 2.2	− 2.2	− 2.3	− 2.3	− 2.3	− 2.3	− 2.4	− 2.4	− 2.4	− 2.4	− 2.4	− 2.5
Esophagus D_mean_	Δ [%]	− 0.8	− 0.5	− 0.4	− 0.3	− 0.2	− 0.1	− 0.1	− 0.1	− 0.1	− 0.1	− 0.1	0.0	0.0	0.0	0.0	0.0	0.0	0.0	− 0.1	− 0.1
	Accum [%]	− 0.8	− 1.3	− 1.7	− 2.0	− 2.1	− 2.3	− 2.4	− 2.5	− 2.5	− 2.6	− 2.7	− 2.7	− 2.8	− 2.8	− 2.8	− 2.8	− 2.8	− 2.8	− 2.8	− 3.0
Cord D_max_	Δ [%]	− 0.7	− 0.6	− 0.5	− 0.4	− 0.3	− 0.3	− 0.2	− 0.2	− 0.2	− 0.1	− 0.1	− 0.1	− 0.1	0.0	0.0	0.0	0.0	0.0	0.0	0.0
	Accum [%]	− 0.7	− 1.3	− 1.8	− 2.1	− 2.4	− 2.7	− 2.9	− 3.1	− 3.3	− 3.4	− 3.5	− 3.6	− 3.7	− 3.7	− 3.7	− 3.7	− 3.6	− 3.6	− 3.5	− 3.6
Heart D_mean_	Δ [%]	− 0.3	− 0.2	− 0.2	− 0.1	− 0.1	0.0	0.0	0.0	0.0	0.0	0.0	0.0	0.0	0.0	0.0	0.0	0.0	0.0	0.0	0.0
	Accum [%]	− 0.3	− 0.6	− 0.7	− 0.9	− 0.9	− 1.0	− 1.0	− 1.0	− 1.0	− 1.0	− 1.0	− 1.0	− 1.0	− 1.0	− 1.0	− 1.0	− 1.0	− 1.1	− 1.1	− 1.1

Prostate		DTF																			
		1.1	1.2	1.3	1.4	1.5	1.6	1.7	1.8	1.9	2.0	2.1	2.2	2.3	2.4	2.5	2.6	2.7	2.8	2.9	3.0
Time	Δ [%]	9.8	9.8	9.8	9.8	9.8	9.8	9.8	9.8	9.8	9.8	9.8	9.8	9.8	9.8	9.8	9.8	9.8	9.8	9.8	9.8
	Accum [%]	9.8	19.6	29.4	39.2	49.1	58.9	68.7	78.5	88.3	98.1	107.9	117.7	127.5	137.3	147.2	157.0	166.8	176.6	186.4	196.2
MF	Δ [%]	11.0	11.0	11.0	11.0	11.0	11.0	11.0	11.0	11.0	11.0	11.0	11.0	11.0	11.0	11.0	11.0	11.0	11.0	11.0	11.0
	Accum [%]	11.0	21.9	32.9	43.9	54.8	65.8	76.8	87.8	98.7	109.7	120.7	131.6	142.6	153.6	164.5	175.5	186.5	197.5	208.4	219.4
CI	Δ [%]	− 0.1	0.2	0.4	0.4	0.4	0.3	0.2	0.1	0.0	− 0.1	− 0.2	− 0.2	− 0.2	− 0.2	− 0.2	− 0.1	− 0.1	− 0.1	− 0.1	− 0.3
	Accum [%]	− 0.1	0.1	0.5	0.9	1.3	1.6	1.8	1.9	1.9	1.8	1.7	1.5	1.3	1.1	1.0	0.8	0.7	0.7	0.5	0.2
HI	Δ [%]	− 8.2	− 5.9	− 4.1	− 2.8	− 1.7	− 1.0	− 0.5	− 0.1	0.1	0.3	0.4	0.5	0.5	0.6	0.6	0.7	0.7	0.6	0.5	0.2
	Accum [%]	− 8.2	− 14.1	− 18.2	− 21.0	− 22.7	− 23.7	− 24.2	− 24.3	− 24.2	− 23.9	− 23.5	− 23.0	− 22.5	− 21.9	− 21.2	− 20.6	− 19.9	− 19.3	− 18.8	− 18.6
Bladdr D_mean_	Δ [%]	− 1.7	− 1.2	− 0.9	− 0.7	− 0.5	− 0.4	− 0.3	− 0.2	− 0.2	− 0.1	− 0.1	− 0.1	− 0.1	0.0	0.0	0.0	0.0	0.0	0.0	− 0.1
	Accum [%]	− 1.7	− 2.9	− 3.8	− 4.5	− 5.0	− 5.4	− 5.6	− 5.8	− 6.0	− 6.2	− 6.3	− 6.4	− 6.4	− 6.5	− 6.5	− 6.5	− 6.4	− 6.4	− 6.4	− 6.5
Rectum D_mean_	Δ [%]	− 4.5	− 3.0	− 1.9	− 1.2	− 0.7	− 0.4	− 0.2	− 0.2	− 0.2	− 0.2	− 0.2	− 0.2	− 0.1	− 0.1	0.0	0.1	0.1	0.1	0.0	− 0.2
	Accum [%]	− 4.5	− 7.6	− 9.5	− 10.7	− 11.4	− 11.8	− 12.0	− 12.2	− 12.4	− 12.5	− 12.7	− 12.9	− 13.0	− 13.1	− 13.1	− 13.0	− 12.9	− 12.7	− 12.7	− 12.9

## Discussion

This study demonstrated that increasing the DTF by 0.1 results in increasing the treatment time by almost 10% when the treatment time for plans with a DTF of 1.0 is normalized to 100%. This relationship was similar for all treatment sites investigated in this study, and the same relationship was observed for the MF. Previous studies have shown that MF has a direct effect on treatment time^2,30,31,39–42^. Generally, a high MF results in an increase in treatment time, although it provides superior dose distribution and lower doses in normal tissues. However, when the MF is small, the delivery time shortens, resulting in poorer dose conformity and homogeneity.

A higher DTF indicates that the leaf opening times become inhomogeneous, leading to an increase in the MF. In other words, a higher DTF allows for a further modulated beam intensity, which could improve the quality of the treatment plan. Our results show that an increase in DTF can improve HI, CI, and OAR doses compared to plans with a DTF of 1.0, except for the CI in the lung cancer case. However, the improvement of indices plateaued at a certain DTF in this study except for the mean doses to both parotid glands in the head and neck plans. Nevertheless, treatment time and MF continued to increase linearly with increasing DTF. This finding indicated that excess DTF leads to an unnecessary increase in treatment time, leading to disadvantages for patients and medical workers. However, the CI markedly deteriorated when the DTF increased above 1.0 in the lung cancer case. Unfortunately, we could not find a clear explanation for this finding; however, this may be caused by a trade-off between the target dose conformity and sparing doses to the surrounding OARs, such as the lung. Another possible reason is that the planning isocenter position could not be set inside the target region for some patients due to the limitation of treatment couch movement in the lateral direction. The relationship between the isocenter position and the target is considered to be crucial for plan quality^43^.

The relationship between the DTF and MF was also evaluated. The MF was approximately 2.0 when the DTF was 1.8, which shows a plateau in plan quality for prostate cancer cases. Nevertheless, this MF is reasonable and lower than that recommended in a previous study^39^. In contrast, when the DTF was within the range of 1.2–1.3 and 1.5–1.6 for head and neck and lung cases, respectively, the MF reached 3.0, which is recognized as a much higher value for head and neck plans^44^. A higher MF leads to greater plan complexity in tomotherapy, potentially leading to unacceptable dose delivery^28,29^. Furthermore, the Radixact unit cannot deliver radiation for treatment plans with an MF of more than 5 due to the machine’s limitation. Therefore, it is necessary to select a DTF with a good balance between treatment time and plan quality. We propose that a DTF of approximately 2.0 is suitable for prostate plans; however, for complex sites such as the head and neck, and lung, a lower DTF (1.3–1.5) should be used at the beginning of treatment planning, considering the treatment time and the improvement of plan quality.

Limitations of this study are that only a small number of patients were examined for each treatment site, and only specific sites were focused on. Increasing the patient numbers in future research could strengthen this study’s conclusions. Another limitation is not to consider changes in planning parameters (FW and pitch) other than DTF, even though combinations of these parameters impacted both delivery efficiency and plan quality improvement^41,45^. Furthermore, the same dose optimization parameters were used for creating the plan with any DTF in this study. However, as seen in Table S1, the residual global objective values did not achieve 0 in any plans, and the rate of change in the values was still small at higher DTF. Additionally, some OAR doses, such as mean doses to the bladder and rectum, plateaued for plans with a large range of DTF. In that case, adjusting the DTF and dose optimization parameters might lead to a change in target conformity and homogeneity and OAR sparing while maintaining the delivery time efficiency. These indicate that our findings are limited to specific datasets. In addition, the percentage difference between the plans with different DTF values may not always be clinically relevant. For example, a 6.9% reduction in the mean dose for both parotid glands when changing the DTF from 1.0 to 3.0 corresponded to a reduction of the dose from 14.7 to 13.6 Gy, resulting in a dose difference of 1.1 Gy. Similarly, a 4.0% reduction in the mean dose to the lung corresponded to a dose reduction of only 0.3 Gy. Therefore, patient-specific selection of the DTF and dosimetric evaluation should be considered in treatment planning in each institution. Nevertheless, due to limited time, determining patient-specific DTF values might be time consuming in clinical practice. Thus, our results could help minimize the time required for fine-tuning DTF values and can serve as a planner’s reference in routine clinical practice. This study may provide a guideline for tomotherapy treatment planning, thus increasing the understanding of how DTF interacts with plan quality and delivery efficiency.

## Conclusions

This study investigated the influence of a new planning parameter provided by RayStation TPS, the DTF, on helical tomotherapy plans for head and neck, lung, and prostate cancers. The results demonstrated that the DTF is a critical parameter for both plan quality and treatment time. Increasing the DTF led to a monotonous increase in the treatment time and MF while improving the dose conformity and homogeneity to the target volume and sparing the OARs. However, in some cases, the positive effect on plan quality was limited to certain DTF levels. Therefore, planners and radiation oncologists need to consider the appropriate trade-off between better plan quality and shorter treatment time when deciding on the adjustment of the DTF.

## Supplementary Information

Supplementary Table S1.

## Data Availability

The data supporting the findings of this study are available from the corresponding author upon reasonable request.
