# Supplementary material for: Impact of delivery time factor on treatment time and plan quality in tomotherapy
Source: Sci Rep. 2023 Jul 27;13:12207. doi: 10.1038/s41598-023-39047-z (PMC10374581; doi:10.1038/s41598-023-39047-z)
Supplement: Supplementary file 1 — Supplementary Table S1. [file 41598_2023_39047_MOESM1_ESM.pdf]

Table S1. Residual global objective values at the end of optimization process

Head & neck	DTF																				
	1.0	1.1	1.2	1.3	1.4	1.5	1.6	1.7	1.8	1.9	2.0	2.1	2.2	2.3	2.4	2.5	2.6	2.7	2.8	2.9	3.0
H01	1.4357	1.6426	1.5256	1.4473	1.3903	1.3317	1.2907	1.4276	1.3910	1.3545	1.4808	1.4440	1.4034	1.3711	1.3431	1.4237	1.3939	1.3659	1.3426	1.4271	1.3997
H02	1.0092	0.9791	0.9585	0.9388	0.9402	0.9507	0.9410	0.9118	0.9101	0.9224	0.9197	0.9154	0.9175	0.9146	0.9132	0.9118	0.9137	0.9124	0.9129	0.9134	0.9152
H03	1.1694	1.0628	1.0128	0.9700	0.9347	0.8981	0.8817	0.8607	0.8443	0.9208	0.9113	0.8877	0.8787	0.8741	0.8490	0.8450	0.9137	0.8909	0.8901	0.8765	0.8680
H04	1.8903	1.7214	1.5893	1.4859	1.4220	1.3697	1.5504	1.4833	1.4321	1.3839	1.5266	1.4834	1.4430	1.4037	1.3820	1.4832	1.4468	1.4271	1.4020	1.4955	1.4632
H05	1.1628	1.0974	1.2822	1.2448	1.1794	1.1415	1.1102	1.2393	1.2101	1.1819	1.1578	1.1247	1.2324	1.1919	1.1816	1.1445	1.2354	1.2137	1.1791	1.1594	1.1407
H06	1.4540	1.2602	1.1335	1.3519	1.2325	1.1464	1.0797	1.2563	1.1964	1.1176	1.2838	1.2410	1.1886	1.3161	1.2697	1.2127	1.3351	1.2690	1.2339	1.1938	1.3044

Lung	DTF																				
	1.0	1.1	1.2	1.3	1.4	1.5	1.6	1.7	1.8	1.9	2.0	2.1	2.2	2.3	2.4	2.5	2.6	2.7	2.8	2.9	3.0
L01	0.5120	0.7545	0.6656	0.5829	0.5267	0.4786	0.6473	0.5965	0.5581	0.5284	0.4914	0.5991	0.5647	0.5263	0.5069	0.6111	0.5832	0.5596	0.5201	0.5034	0.5963
L02	0.1302	0.1291	0.1302	0.1305	0.1316	0.1426	0.1324	0.1407	0.1332	0.1398	0.1331	0.1330	0.1337	0.1381	0.1383	0.1390	0.1393	0.1388	0.1387	0.1387	0.1393
L03	0.9305	0.6532	0.4094	0.2938	0.2773	0.2598	0.2517	0.2414	0.2338	0.2312	0.2213	0.2032	0.2032	0.2024	0.2141	0.2007	0.2082	0.2087	0.1987	0.2059	0.1954
L04	0.1933	0.1821	0.1743	0.1700	0.1647	0.1618	0.1586	0.1570	0.1559	0.1553	0.1547	0.1549	0.1544	0.1531	0.1524	0.1520	0.1510	0.1502	0.1504	0.1497	0.1502
L05	0.1604	0.1553	0.1570	0.1586	0.1606	0.1603	0.1602	0.1627	0.1602	0.1601	0.1635	0.1635	0.1604	0.1595	0.1602	0.1620	0.1646	0.1635	0.1634	0.1647	0.1642
L06	0.3331	0.2923	0.2697	0.2498	0.2427	0.2306	0.2305	0.2174	0.2260	0.2239	0.2204	0.2432	0.2370	0.2071	0.2046	0.2034	0.2199	0.2207	0.2182	0.2149	0.2128
L07	0.2027	0.1763	0.1719	0.1494	0.1476	0.1473	0.1472	0.1460	0.1471	0.1461	0.1479	0.1483	0.1512	0.1550	0.1542	0.1545	0.1542	0.1534	0.1534	0.1550	0.1545
L08	0.4002	0.3505	0.3158	0.2917	0.2675	0.2581	0.3036	0.2891	0.2711	0.2682	0.2625	0.2569	0.2510	0.2466	0.2719	0.2642	0.2562	0.2510	0.2508	0.2412	0.2406
L09	0.1659	0.1556	0.1474	0.1393	0.1300	0.1382	0.1330	0.1264	0.1260	0.1273	0.1269	0.1256	0.1262	0.1279	0.1279	0.1263	0.1270	0.1264	0.1255	0.1278	0.1251

Prostate	DTF																				
	1.0	1.1	1.2	1.3	1.4	1.5	1.6	1.7	1.8	1.9	2.0	2.1	2.2	2.3	2.4	2.5	2.6	2.7	2.8	2.9	3.0
P01	1.0136	0.4994	0.4033	0.3428	0.2985	0.3566	0.3257	0.2965	0.3471	0.3157	0.3973	0.3384	0.3162	0.3601	0.4034	0.3812	0.3638	0.4017	0.3802	0.4147	0.3973
P02	0.5725	0.4129	0.3452	0.3084	0.3687	0.3209	0.2893	0.2630	0.2362	0.2814	0.2622	0.2471	0.2847	0.2691	0.2531	0.2898	0.2734	0.2603	0.2942	0.2771	0.2662
P03	0.5663	0.5878	0.4977	0.4385	0.3931	0.3468	0.3145	0.2896	0.3372	0.3161	0.2960	0.3424	0.3236	0.3040	0.3434	0.3235	0.3119	0.2968	0.3309	0.3192	0.3038
P04	0.5772	0.4791	0.4134	0.4835	0.4291	0.3846	0.3521	0.4054	0.3715	0.3454	0.3943	0.3721	0.4178	0.3921	0.3698	0.4095	0.3905	0.3707	0.4075	0.3905	0.3757
P05	0.6667	0.6470	0.5395	0.4623	0.4045	0.4607	0.4193	0.3716	0.3383	0.3102	0.3546	0.3325	0.3774	0.3572	0.3348	0.3743	0.3548	0.3378	0.3709	0.3539	0.3854
P06	0.3556	0.2598	0.3355	0.2940	0.2564	0.2256	0.2053	0.2568	0.2379	0.2208	0.2060	0.2518	0.2350	0.2771	0.2622	0.3006	0.2909	0.2741	0.2583	0.2931	0.2794
P07	1.1168	0.8645	0.5667	0.4887	0.4292	0.3820	0.4412	0.4020	0.3649	0.3326	0.3768	0.3560	0.3985	0.3754	0.3538	0.3939	0.3735	0.3544	0.3837	0.3714	0.3557
P08	0.5217	0.4168	0.3584	0.3068	0.3680	0.3225	0.2871	0.2602	0.2397	0.2896	0.2653	0.2511	0.2902	0.2737	0.2581	0.2955	0.2813	0.2652	0.2999	0.2866	0.2750
P09	0.6776	0.6141	0.5041	0.4428	0.3768	0.3305	0.2963	0.2679	0.3177	0.2981	0.2751	0.3224	0.2981	0.2801	0.2647	0.3041	0.2897	0.2766	0.3133	0.2996	0.3336
P10	0.5529	0.4326	0.3547	0.4281	0.3664	0.3188	0.2920	0.2635	0.3093	0.2802	0.2610	0.2435	0.2877	0.2697	0.2577	0.2931	0.2753	0.2635	0.2511	0.2826	0.2708
